# Case of Frequent Uterine Hemorrhagy, with Incessant Discharge of the Liquor Amnii, Four Months Previous to Labour; with Some Remarks on the Ergot of Rye

**Published:** 1830-02

**Authors:** J. H. Burgess

**Affiliations:** Surgeon.


### UTERINE HEMORRAGY.

*Case of frequent Uterine Hemorrhagy, with incessant Discharge of the Liquor Amnii, four months previous to Labour; with some Remarks on the Ergot of Rye.* By J. H. Burgess, Esq. Surgeon.

Mrs. B., ætatis thirty-one, of spare and delicate habit, already mother of three children, was attacked, on the evening of the 10th of June, 1829, with slight uterine hemorrhagy, and discharge of the liquor amnii to a considerable quantity, being (as she supposed) about three months advanced in pregnancy. It was naturally expected that miscarriage would follow in a short time. She was ordered astringent medicines, perfect quiet in the horizontal position, an opiate, and the frequent application of cooling lotions to the pubes and labiæ. There was no pain, nor could she account for such appearances, otherwise than from her having walked, the day preceding, a distance of three or four miles.

The treatment had the desired effect as regarded the hemorrhage, but the discharge of liquor amnii continued unabated.

All went on well until September 10th, when violent hemorrhagy returned. Mrs. B. was then desired on no occasion to leave her bed without permission; and the usual medicines being given, such as a full dose of Tinct. Opii, followed by Mist. Infus. Rosæ cum Acid. Sulph. dilut. &c. together with the lotion as before, the hemorrhage was again arrested.

On the 11th, Mrs. B. complained of considerable bearing down, with a sensation as though the fœtus would be instantly expelled; but, on examination per vaginam, it was discovered to arise from a large coagulum, completely filling up the parts. Fifty drops of Tinct. Opii were immediately given, and the coagulum suffered to remain, fearing its removal might endanger a return of hemorrhage. Shortly after taking the opiate, it produced sleep of two hours' continuance; when Mrs. B. awoke much refreshed, and free from hemorrhage, yet the liquor amnii continued draining away.

13th.—Pulse ninety-six, and feeble; bowels constipated; slight headach; heat of the skin; coated tongue, with occasional nausea.—Ordered Haust. Salin. in statu effervescentiæ tertiis horis, et Haust. Ol. Ricini cum Aqua Cinnamomii statim sumendus. The coagulum came off this morning. In the evening Mrs. B. appeared much better: has had three offensive feculent evacuations. Liquor amnii still discharging.

14th.—Appears better; no hemorrhage; liquor amnii still escaping; urine natural.

No material alteration occurred for many days. Liquor amnii continued flowing away. The patient was kept quiet, and occasional aperients were given. On the 24th, slight hemorrhagy without pain. It ceased spontaneously in about half an hour.

25th.—Was called up in the night, Mrs. B. having a return of hemorrhagy to an alarming extent; no pain. An examination per vaginam was instituted, when the os uteri was found apparently closed. The hemorrhage was arrested by the before-mentioned means, conjoined with a full dose of Tinct. Opii.

Mrs. B. went on from this period with unceasing discharge of the liquor amnii, and the occasional use of aperient medicines, until October the 6th, when I was again called in the night, from her having a return of hemorrhagy, still without pain. She fell into a state of syncope immediately on the application of the lotion to the pubes, remaining so for the space of two minutes. The discharge of blood, as is usual during fainting, had ceased, and did not now again return. An examination was made per vaginam, hoping to find the os internum gradually dilating, so as to admit of delivery; but no such occurrence had taken place, therefore it became impracticable.

7th.—Exhaustion appeared so great, it was deemed necessary to administer slightly cordial medicines, with Tr. Opii, and an occasional tablespoonful of wine. Liquor amnii still escaping; urine natural.

8th.—Somewhat better, but the uterine liquor still draining away.

9th.—As yesterday. Slept well during the night.

10th.—Bowels not moved since the 8th.—℞. Haust. Oleos. statim, et rep. dosis si opus sit.

11th.—The bowels moved twice, bringing off a considerable quantity of hardened feces.

12th.—Complains of headach, preceded by slight rigors, followed by considerable heat of skin. Pulse ninety-six, and bounding; bowels not acted on to-day.—Ordered Haust. Salin. cum Magn. Sulph. ʒi.; Tr. Digitalis gtt. vi. quartis horis sumend, donec alvus soluta fuerit.

13th.—Copious and offensive evacuations from the bowels, much to the relief of the patient. Pulse eighty; liquor amnii escaping as usual; urine natural.

14th.—Much better. Still desired not to leave her bed on any account.

All went on as on the 8th instant, with the occasional administration of aperient medicines, until the 23d, when I was sent for in haste, Mrs. B. appearing to her attendants in great danger. On arrival, found the countenance and general surface extremely pale; pulse frequent and feeble. Tinct. Opii gtt. iv. were immediately given in a little water, but was soon rejected by vomiting. Symptoms of labour were evidently indicated by slight pains occurring about every ten minutes, together with a great flow of the liquor amnii and violent hemorrhagy. This was about one o'clock P.M. An examination per vaginam was instantly made, and the os internum found dilated nearly the size of a shilling; the hemorrhage at this moment profuse. The patient was placed on her left side, with hips much elevated, and, during each pain, a cautious effort was made to dilate the os internum, by means of the first and second fingers of the right hand. It was found, on the first effort to pass the finger within the uterus, that the placenta was firmly attached to its cervix; and, while pressing or drawing the os internum, (which was very rigid, more so than it generally is in cases of uterine hemorrhagy,) at its anterior part, toward the pubis, the bleeding was checked; and, keeping up such pressure, I waited some time, hoping the parturient efforts might increase; but, instead of which, the pains became gradually weaker and less frequent. I then determined to make trial of the ergot of rye, in decoction, of the following strength: ℞. Ergot contus. ʒi. coque in Aqua ℥vi. ad ℥iij.; of which I gave ℥iss. and repeated the dose in ten minutes. Its effects were in this case more conspicuous than any other in which I have ever made trial of it, although I have frequently used it with marked success. In twelve minutes from the administration of the first dose, its action became manifest: to such a degree were the expulsatory efforts increased, that in about five minutes more, the os internum was sufficiently dilated for the introduction of the hand; when it was cautiously and steadily passed by the edge of the detached placenta, and the head instantly felt resting above it, (the hemorrhage at this time profuse.) While endeavouring to reach the feet, with a view of expediting the delivery, such powerful contraction of the uterus came on, that it was found necessary to withdraw the hand, fearing such violent efforts might endanger laceration of the uterus, while acting on it. The moment the hand was withdrawn into the vagina, the fœtus followed (from the long continuance of such expulsatory effort), and was happily delivered.

After applying a ligature to the funis, and removing the child, which was then alive, I made an external examination of the abdomen, when I instantly suspected the existence of another child; and, on examining per vaginam, found the membranes of a second fœtus brought down to the os internum. A pain coming on immediately, I ruptured the membrane, and a second child, together with both placentæ, were expelled instantly, with the greatest imaginable force.

The hemorrhage immediately ceased; and, on examining the placenta of the first child, it was found to have been separated to about one half of its area, and a considerable part cicatrised; clearly proving the cause of such repeated and alarming hemorrhages.* At the moment of writing this, Mrs. B. appears going on well.

The above case is, I believe, one of the most serious and perplexing that can possibly fall to the lot of any man to treat, or his patient to bear; and, from my not having seen, or even read of, a similar case in all its bearings, particularly as regards the continued draining of the liquor amnii for such an extraordinary period as four months, I am induced to give it publicity. In the cases which have fallen under my notice of premature rupture of the membranes, labour has invariably commenced within five days; but, in this case, the first discharge of the liquor amnii took place on the 10th day of June: labour did not commence until October 23d following; being, I believe, an unparalleled period of 135 days!

In regard to the use of the ergot of rye, I have found it, in many cases of uterine hemorrhagy and protracted labour, a medicine of the greatest use and importance; but caution in distinguishing the proper period and case for its employment is indispensable, not only as regards the safety of the mother, but the child. In all first cases, unaccompanied with hemorrhage, I should not be disposed to have recourse to it, until the os internum was fully dilated, or the parts in a proper state for its employment. In protracted cases of natural labour, where the pains appear inefficient, and no deformity exists, yet the os internum rigid, and not fully, or nearly so, dilated, (unless I found other symptoms of alarm,) I should wait patiently for the complete dilatation of the os internum before I tried the ergot; there being, I conceive, imminent danger of rupturing even the cervix uteri, from the violence of the throes when its action becomes fully developed. Yet, in all cases of uterine hemorrhagy, arising from causes before enumerated, where the life of the mother is in jeopardy, I believe, from the experience I have had, it may be administered, even before the complete dilatation of the os internum, with a happy result.

It has been said that the violent contractions of the uterus (after the administration of the ergot) cannot be restrained: this I am inclined to doubt. I believe opium to possess a sufficient antispasmodic influence to arrest its action in a

* Query, whether the cause of delay in the commencement of labour may not be attributable to the continued distention of the uterus in consequence of twins, thereby allowing the constant escape of the uterine fluid from one membrane, while the second retained its natural magnitude.—J. H. B.

few minutes, especially after the expulsion of the contents of the uterus; as, in the case here detailed, the administration of fourteen drops of tincture of opium relieved the patient in ten minutes of the most violent after-pains. I have seldom found it necessary, as in this case, to repeat the dose; but would wish it to be clearly understood that, (as far as my judgment serves me,) in all cases where a trial of the ergot is had recourse to, the dose should be repeated in ten or fifteen minutes, if its effects are not fully established. Ample experience has taught me that the ergot is a very valuable medicine in the hands of the accoucheur who applies it with judgment and discrimination.

*Glastonbury; November*, 1829.

